# Supplementary material for: DMRforPairs: identifying Differentially Methylated Regions between unique samples using array based methylation profiles
Source: BMC Bioinformatics. 2014 May 15;15:141. doi: 10.1186/1471-2105-15-141 (PMC4046028; doi:10.1186/1471-2105-15-141)
Supplement: Additional file 2 — DMRforPairs output for the comparison of A431-MCF7 and NA17018-NA17105. Please start from the HTML files in each folder. Available via the BMC Bioinformatics website. [file 1471-2105-15-141-S2.zip › 1394847754114233_MOESM2_ESM/A431_MCF7/figures/4505.pdf]

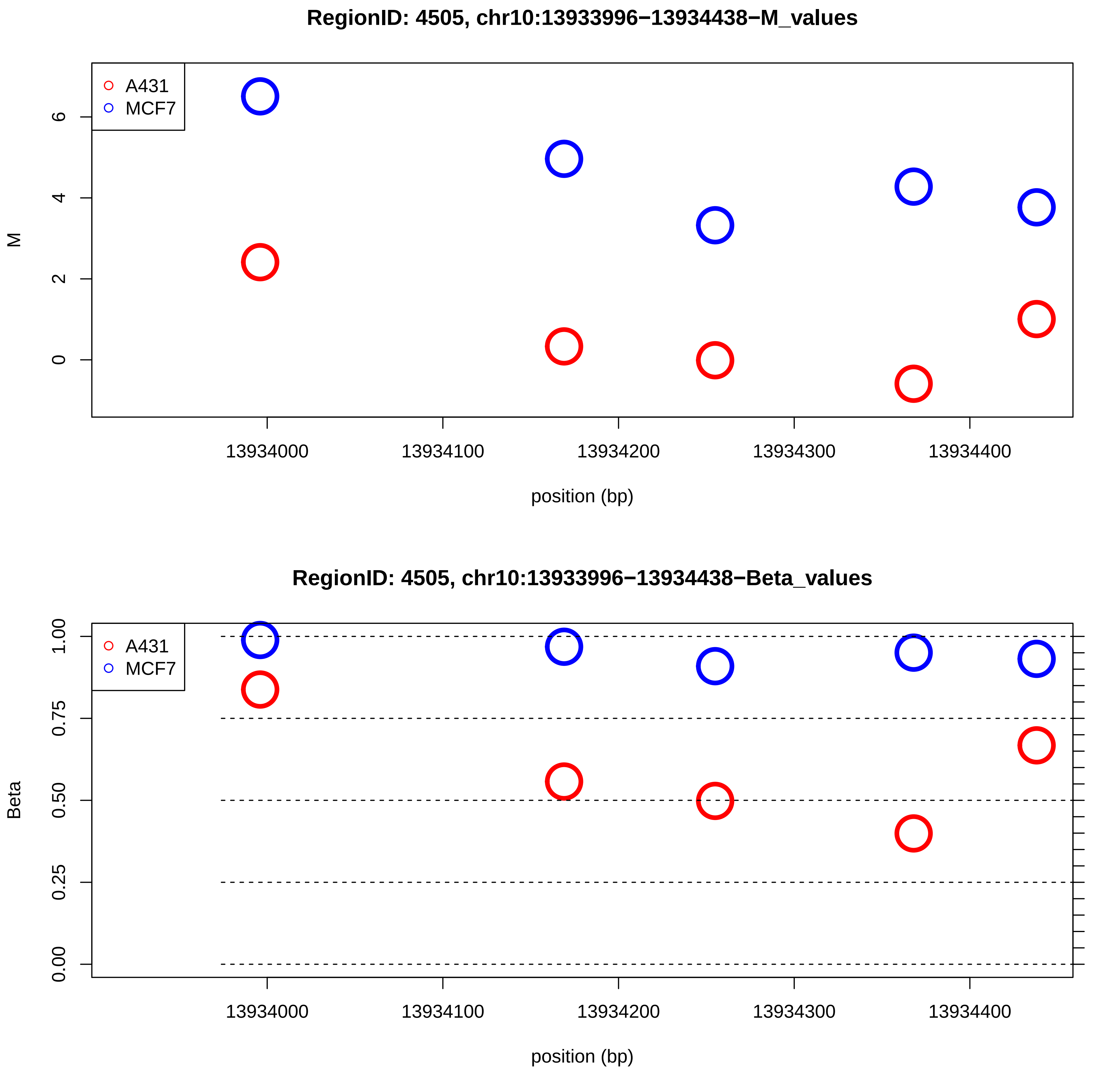

RegionID: 4505, chr10:13933996−13934438−M_values
A431
MCF7
M
0
2
4
6
13934000
13934100
13934200
13934300
13934400
position (bp)
RegionID: 4505, chr10:13933996−13934438−Beta_values
A431
MCF7
Beta
0.00
0.25
0.50
0.75
1.00
13934000
13934100
13934200
13934300
13934400
position (bp)

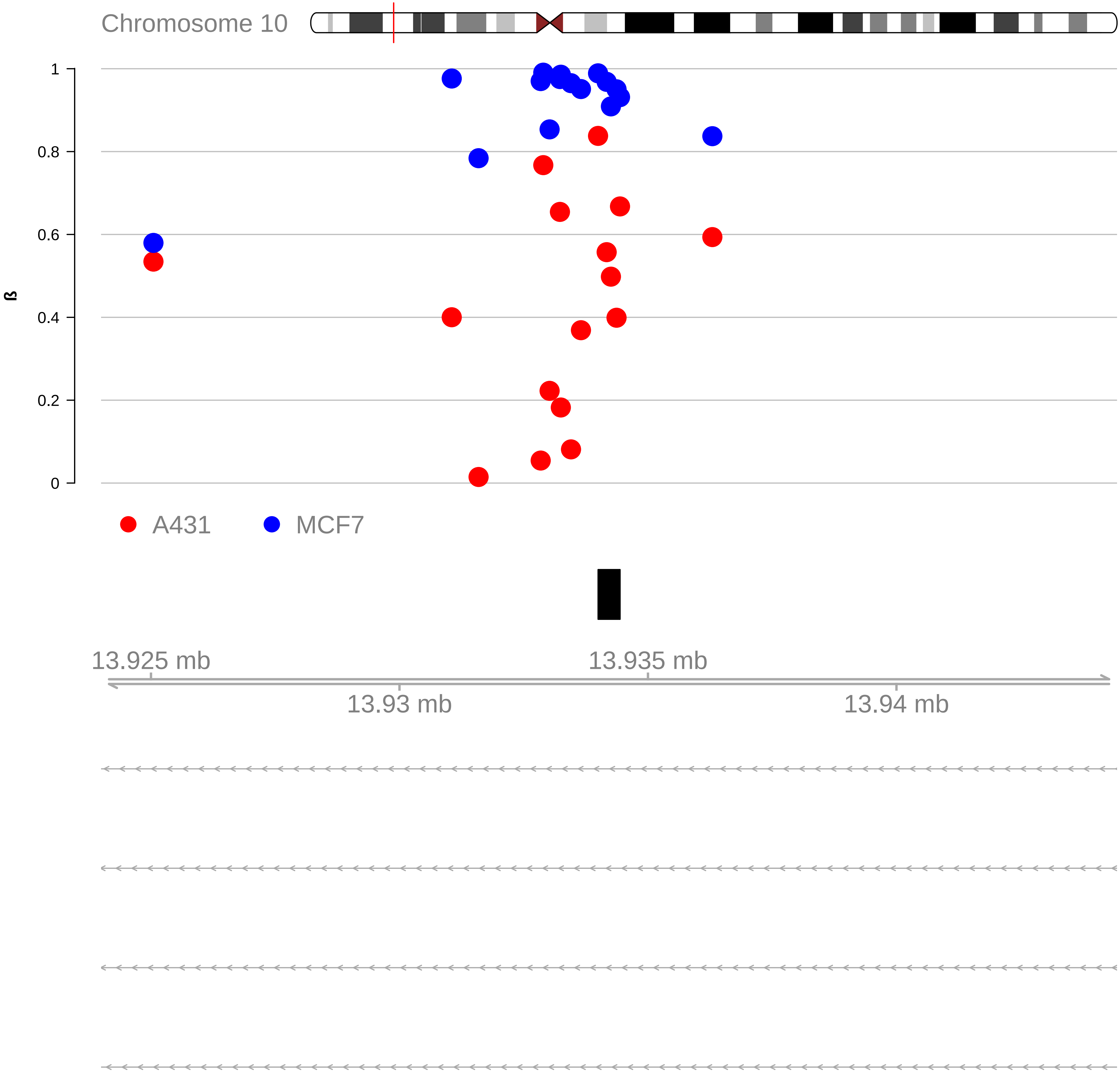

Chromosome 10
1
0.8
0.6
0.4
0.2
0
ß
A431
MCF7
13.925 mb
13.935 mb
13.93 mb
13.94 mb
